# Nicotine Induces the Up-regulation of the α7-Nicotinic Receptor (α7-nAChR) in Human Squamous Cell Lung Cancer Cells via the Sp1/GATA Protein Pathway*

**DOI:** 10.1074/jbc.M113.501601

**Published:** 2013-10-02

**Authors:** Kathleen C. Brown, Haley E. Perry, Jamie K. Lau, Dennie V. Jones, Joseph F. Pulliam, Brent A. Thornhill, Clayton M. Crabtree, Haitao Luo, Yi. Charlie Chen, Piyali Dasgupta

**Affiliations:** From the ‡ Department of Pharmacology, Physiology, and Toxicology, Joan C. Edwards School of Medicine, Marshall University, Huntington, West Virginia 25755,; § Division of Oncology and; ¶ Division of Pathology, Department of Medicine, Markey Cancer Center, University of Kentucky, Lexington, Kentucky 40536, and; the ‖ Department of Biology, Alderson-Broaddus University, Philippi, West Virginia 26416

**Keywords:** GATA, Lung Cancer, Nicotinic Acetylcholine Receptors, Proliferation, Sp1

## Abstract

Nicotine, the addictive component of cigarettes, promotes lung cancer proliferation via the α7-nicotinic acetylcholine receptor (α7-nAChR) subtype. The present manuscript explores the effect of nicotine exposure on α7-nAChR levels in squamous cell carcinoma of the lung (SCC-L) *in vitro* and *in vivo*. Nicotine (at concentrations present in the plasma of average smokers) increased α7-nAChR levels in human SCC-L cell lines. Nicotine-induced up-regulation of α7-nAChR was confirmed *in vivo* by chicken chorioallantoic membrane models. We also observed that the levels of α7-nAChR in human SCC-L tumors (isolated from patients who are active smokers) correlated with their smoking history. Nicotine increased the levels of α7-nAChR mRNA and α7-nAChR transcription in human SCC-L cell lines and SCC-L tumors. Nicotine-induced up-regulation of α7-nAChR required GATA4 and GATA6. ChIP assays showed that nicotine induced the binding of GATA4 or GATA6 to Sp1 on the α7-nAChR promoter, thereby inducing its transcription and increasing its levels in human SCC-L. Our data are clinically relevant because SCC-L patients smoked for decades before being diagnosed with cancer. It may be envisaged that continuous exposure to nicotine (in such SCC-L patients) causes up-regulation of α7-nAChRs, which facilitates tumor growth and progression. Our results will also be relevant to many SCC-L patients exposed to nicotine via second-hand smoke, electronic cigarettes, and patches or gums to quit smoking.

## Teaser

**Background:** Nicotine promotes the proliferation of human squamous cell lung cancer (SCC-L) via the α7-nicotinic receptor (nAChR).

**Results:** Nicotine increases α7-nAChR expression via transcriptional mechanisms involving Sp1 and GATA proteins.

**Conclusion:** Nicotine-induced up-regulation of α7-nAChR accelerates the growth of human SCC-L.

**Significance:** SCC-L patients exposed to nicotine display fast growing lung tumors and worse clinical outcomes.

## Introduction

Squamous cell carcinoma of the lung (SCC-L)^4^ is a form of non-small cell lung cancer that predominantly originates from the bronchial epithelial cell lining, centrally located in large airways. It is the second most prevalent form of lung cancer, accounting for 30% of all primary pulmonary malignancies (1, 2). Epidemiologic and laboratory-based studies have established a causal relationship between cigarette smoking and SCC-L (1, 3). Although cigarette smoke is a mixture of thousands of compounds, nicotine is the addictive component of cigarettes. Data from cell culture and animal models have shown that nicotine stimulates the growth of squamous cell carcinoma (4, 5). In addition, nicotine promotes angiogenesis invasion and metastasis of human non-small cell lung cancers (6, 7).

The biological effects of nicotine are mediated by nicotinic acetylcholine receptors (nAChRs) on human lung cancer cells (8, 9). Non-neuronal nAChRs are pentameric proteins composed of a combination of two different types of subunits (α and β) or five copies of the same α subunit symmetrically arranged around a central ion pore. The homomeric α7-nAChR (composed of five α-subunits) mediates the proliferative, pro-angiogenic, and pro-metastatic effects of nicotine in human non-small cell lung cancers (7, 10). Several types of human lung cancers, including lung adenocarcinoma, SCC-Ls, and small cell lung cancers, have been found to express α7-nAChRs (5, 7). The expression of α7-nAChRs is regulated by a wide variety of extracellular stimuli including glucocorticoids, growth factors, tobacco carcinogens (*e.g.* NNK), and nicotine itself (11–13).

Several convergent studies have shown that nicotine exposure up-regulates the expression of nAChRs in neuronal cells (14, 15). However, these studies have explored the effects of nicotine exposure on α4/β2 nAChRs in the brain (14, 16). There are relatively fewer research papers that have studied the effect of nicotine exposure on α7-nAChRs in non-neuronal cells (17–19). In addition, the mechanisms underlying the increased levels of α7-nAChRs (in response to nicotine) in non-neuronal cells remain to be fully understood.

Studies by Lam *et al.* (20) have shown that nicotine caused robust up-regulation of α7-nAChR mRNA in human bronchial epithelial cells. Nicotine increased the levels of α7-nAChR by transcriptional mechanisms involving the Sp1-GATA2 pathway in human keratinocytes (19). Taken together, these observations suggest that nicotine can increase the levels of α7-nAChR by transcriptional mechanisms in non-neuronal cells.

The α7-nAChR promoter has several binding sites for Sp1 (21–23). Arredondo *et al.* (19) showed that long term exposure to nicotine increased α7-nAChR in human keratinocytes by the Sp1-GATA2 pathway. They performed siRNA experiments and electrophoretic mobility shift assays which showed that the GATA2 transcription factor was bound to the α7-nAChR promoter upon nicotine treatment (19). They inferred that nicotine-induced α7-nAChR up-regulation was mediated by the Sp1-GATA2 pathway in human keratinocytes. It is known that the Sp1 protein can directly associate with the GATA family of transcription factors to regulate gene expression (24, 25). Based on these results we decided to investigate whether the Sp1-GATA pathway was responsible for nicotine-induced up-regulation of α7-nAChR in human SCC-Ls.

Data from several laboratories have shown that α7-nAChRs do not undergo long term inactivation after chronic exposure to nicotine at levels that are present in the plasma of heavy and light smokers (16, 26, 27). Chernyavsky (28) have shown that the biological activity of nAChRs in non-neuronal cells can be attributed to both ion channel-dependent and ion channel-independent events. The ion channel-independent events include activation of protein kinases, second messengers, and transcription factors (28). Taken together, these observations suggest that a large fraction of α7-nAChRs are biologically functional during sustained nicotine exposure (10).

In the present manuscript we show that nicotine up-regulates α7-nAChR expression in human SCC-L cells and in chicken chorioallantoic membrane models. Similarly, the levels of α7-nAChR in human SCC-L tumors isolated from patients (who are active smokers) correlate with their smoking history and amount of cigarette consumption. Luciferase assays revealed that nicotine increased transcription of α7-nAChR in human SCC-Ls. RNAi experiments showed that nicotine-induced up-regulation of α7-nAChR was mediated by GATA4 and GATA6. Finally, chromatin-immunoprecipitation (ChIP) assays demonstrated that nicotine induced the recruitment of Sp1-GATA4 or Sp1-GATA6 complexes on the α7-nAChR promoter, inducing its transcription and increasing its expression in human SCC-Ls.

The reason we decided to study the mechanisms underlying nicotine-induced up-regulation of α7-nAChR is that it may have considerable implications in the pathophysiology of human lung cancers. This is especially true of human SCC-L whose development is closely associated with smoking habits, more so than other non-small cell lung cancers (3, 29). People usually smoke for decades before being diagnosed with SCC-Ls. Previous studies show that ∼30% of smokers with lung cancer continue to smoke after their diagnosis (29, 30). In addition, many SCC-Ls patients are exposed to secondhand smoke, electronic cigarettes, or nicotine patches/gums. It can be envisaged that such long term exposure of human SCC-L tumors to nicotine increases the expression of α7-nAChR on the lung tumor, thereby facilitating its progression and eventual metastasis. Therefore, the study of molecular mechanisms underlying nicotine-induced up-regulation of α7-nAChRs in SCC-L is clinically relevant and will increase our understanding of the role of nAChRs in human lung cancers.

## EXPERIMENTAL PROCEDURES

### 

#### 

##### Reagents, Antibodies, and Constructs

Nicotine, atropine, and α-bungarotoxin were purchased from Sigma. The nAChR antagonist methyllycaconitine was obtained from Tocris Biosciences (Ellisville, MO).

GATA1–6 polyclonal antibodies and Sp1 monoclonal antibody were obtained from Santa Cruz Biotechnology (Santa Cruz, CA). The secondary donkey-anti-rabbit and rabbit-anti-mouse antibodies were obtained from Thermo Scientific, Rockford, IL. Polyclonal GAPDH antibody was obtained from Trevigen, Inc. (Gaithersburg, MD). The bovine α7-nAChR-promoter-Luciferase construct was a kind gift from Dr. M. Criado, Instituto de Neurociencias de Alicante, Universidad Miguel Hernández-Consejo Superior de Investigaciones Científicas, Alicante, Spain.

##### Cell Lines and Cell Culture

The human SCC-L cell lines NCI-H520, NCI-H226, and SK-MES-1 (hereinafter referred to as H520, H226, and SK-MES) were purchased from the ATCC (Manassas, VA). H520 and H226 were cultured in RPMI 1640 medium (Mediatech Inc., Manassas, VA) supplemented with 2 mm glutamine, 10 mm HEPES, 1 mm sodium pyruvate, 4.5 g/liter glucose, 1.5 g/liter sodium bicarbonate, 100 units/ml penicillin, 50 μg/ml streptomycin, and 10% fetal bovine serum (FBS). SK-MES was grown in minimum essential medium with Earle's salts (EMEM) containing non-essential amino acids, 1 mm sodium pyruvate, 100 units/ml penicillin, 50 μg/ml streptomycin, and 10% FBS. These cell lines were authenticated by the ATCC Cell Authentication service using Short Tandem Repeat (STR) profiling techniques (data not shown).

##### Human SCC-L Tumor Samples

**FIGURE 1.**
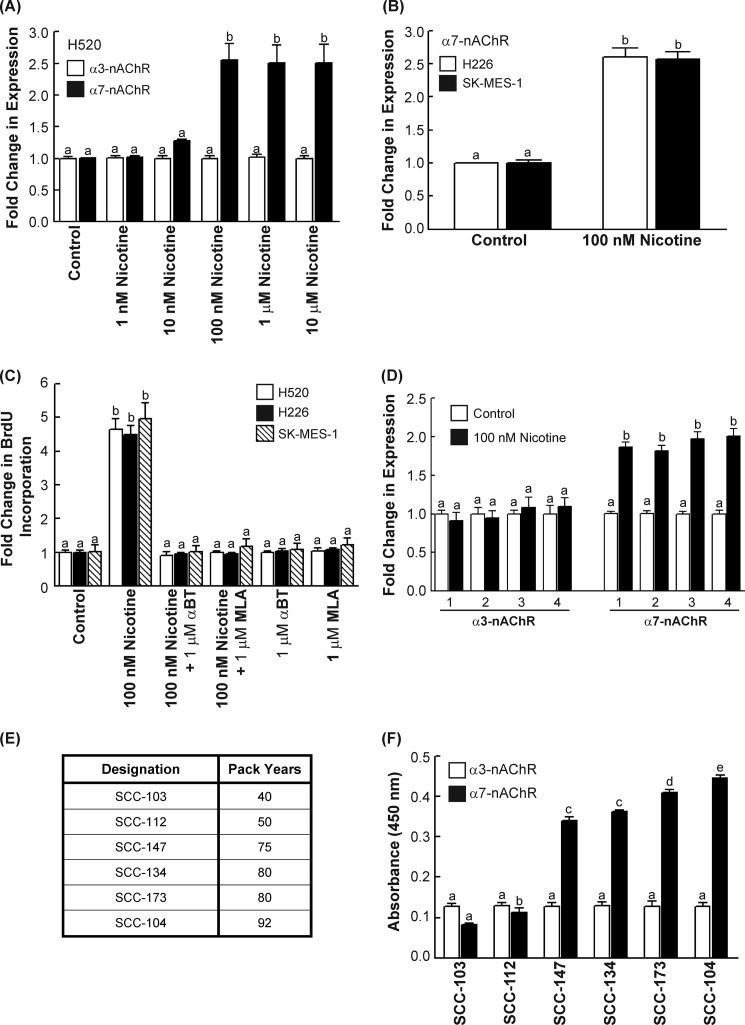
**Nicotine increased α7-nAChR expression in human SCC-L *in vitro* and *in vivo*.** *A*, nicotine up-regulated the levels of α7-nAChRs (*black bars*) in a concentration-dependent manner in H520 human SCC-L cells in 96 h. However, nicotine did not affect the levels of α3-nAChRs (*white bars*). *B*, the treatment of H226 and SK-MES human SCC-Ls with 100 nm nicotine produced robust increase in α7-nAChR levels in both these cell lines at 96 h. *C*, the proliferative effects of nicotine in H520, H226, and SK-MES human SCC-L cells was abrogated by the α7-nAChR antagonists α-bungarotoxin (α*-BT*) and methyllycaconitine (*MLA*), showing that nicotine-induced proliferation of human SCC-L cells was mediated via α7-nAChR. *D*, ELISAs were used to measure α7-nAChR expression in four of the chicken CAM tumors (*marked 1–4*). Nicotine had no effect on the levels of α3-nAChRs (*left panel*), whereas it robustly increased the levels of α7-nAChRs in the nicotine-treated H520 chicken CAM tumors (*right panel*). *E*, a table depicting the smoking history of six SCC-L tumors isolated from patients. *F*, ELISAs showed that the level of α7-nAChR (*white bars*) in these tumors was closely correlated to the smoking history of these patients. The expression of α3-nAChR (*black bars*) was not altered in the SCC-L tumor samples. The assay was completed in duplicate, and the whole experiment was performed two independent times. Results indicated by a different letter are significantly different (*p* < 0.05*).*

Fresh-frozen human SCC-L tissues (*n* = 6) from patients who were active smokers were obtained from the Biospecimen Core Program, Markey Cancer Center, Lexington, University of Kentucky. The use of all SCC-L tumor tissues was approved by the Markey Biospecimen Utilization committee, Markey Cancer Center, Lexington, University of Kentucky. The smoking history of the patients is described in Fig. 1*E*.

##### Lysates and Western Blotting

Lysates for each cell line were made using the Nonidet P-40-based lysis protocol (31, 32). Cells were lysed with M2 lysis buffer as described previously (31, 32). The relative expression of the indicated proteins was analyzed by Western blotting using standard protocols (31, 32). The results of the Western blotting assays were quantitated by ImageJ 1.46p (National Institutes of Health, Bethesda, MD).

Cell lysates were prepared from frozen human SCC-L tumor tissues as described elsewhere (31, 32). The tissues were incubated with 1 ml of ice-cold red blood cell lysis buffer (0.14 m NH_4_Cl and 0.017 m Tris-HCl, pH 7.2) on ice for 5 min. The cells were washed three times in red blood cell lysis buffer until red blood cells were no longer visible. The cell pellet was resuspended in one packed cell volume of M2 lysis buffer. The rest of the protocol was the same as described for preparing lysates from cultured cells.

##### nAChR ELISA Assays

The expression of α7-nAChR and α3-nAChR subunits in human SCC-L cell lines was analyzed by using ELISA kits (Antibodies Online Inc., Atlanta, GA). Each of these assays was completed in duplicate, and the whole experiment was performed two independent times for each cell line.

##### Bromodeoxyuridine (BrdU) Assays

H520 human SCC-Ls were plated in 8-well chamber slides at a density of 10^4^ cells/well. After overnight incubation at 37 °C, the medium of the cells was changed to RPMI containing 2.5% FBS in the presence or absence of 100 nm nicotine. This was designated as day 0. On days 2, 4, and 6, the old medium was aspirated, and new medium containing 100 nm nicotine was added. The rate of BrdU incorporation was measured using a BrdU Labeling and Detection Kit II (Roche Applied Science) according to the manufacturer's instructions. The number of positive cells on day 0 was assumed to be 1. Increases in the number of cells in BrdU-labeled cells (as a result of nicotine treatment) were calculated as a -fold increase of the number of BrdU-positive cells on day 0 (31). Each sample was tested in duplicate, and the BrdU assay was performed two independent times.

##### Chicken Chorioallantoic Membrane (CAM) Assay

H520 cells (3.0 × 10^6^) were suspended in a 1:1 (v/v) solution of serum-free medium and Matrigel (BD Biosciences). This cell suspension was treated with 100 nm nicotine at 4 °C for 30 min and subsequently applied to the 9-day-old chicken embryo CAM as described previously (31). The chicken embryos were incubated for 7 days. After incubation, the H520 tumor implants were removed and weighed. A total of seven eggs were assayed for each group. Cell lysates were made from the above H520 tumors grown on CAM using T-Per tissue lysis buffer according to manufacturer's protocol (Thermo Scientific).

##### RNA Extraction and Real Time PCR

H520 and H226 human SCC-L cells were grown to 50% confluence. Subsequently, the medium was aspirated, and the cells were resuspended in RPMI containing 2.5% FBS in the presence or absence of 100 nm nicotine for 72 h at 37 °C. Total RNA was isolated using the RNeasy Plus Mini kit (Qiagen Sciences Inc., Valencia, CA). This kit was also used to isolate total RNA from human SCC-L tumors isolated from patients. The cDNA was synthesized using the iScript cDNA Synthesis kit (Bio-Rad) (31). Data were normalized using 18 S rRNA as the internal control, and the -fold change in the expression levels was determined relative to the untreated cells (31). The α7-nAChRs primer sequences and PCR conditions are described in Lam *et al.* (20). Real-time PCR was performed on a Bio-Rad iCycler (Bio-Rad).

##### Luciferase Assays

H520 human SCC-Ls were transfected with 2 μg of the construct α7-promoter (bovine)-LUC using FuGENE HD transfection reagent (Roche Applied Science) according to manufacturer's protocols (11). The transfection efficiency was measured by cotransfection with 2 μg of pRL construct containing *Renilla reniformis* luciferase gene (Promega, Madison, WI). The empty vector pGL3-basic (Promega) was used for the mock-transfected cells (33). Twenty-four hours after transfection the medium was aspirated, and the cells were washed once in 1× PBS. Subsequently, the cells were incubated in RPMI supplemented with 2.5% FBS containing 100 nm nicotine for 48 h. Cell lysates were prepared, and the luciferase activity was measured using a Dual-Glo Luciferase assay kit (Promega). Relative luciferase activity was defined as the mean value of the firefly luciferase/renilla luciferase ratios obtained from two independent experiments.

##### siRNA Transfection and Assays

Chemically synthesized, double-stranded GATA4 siRNA and GATA6 siRNA were purchased from Santa Cruz Biotechnologies. A non-targeting siRNA (Santa Cruz Biotechnologies) was used as a control-siRNA for the transfection experiments (34, 35). A second independent set of GATA4 siRNA and GATA6 siRNA was obtained from Ambion (Grand Island, NY). The transfection experiments were performed in H520 and H226 human SCC-Ls. The transfection of 75 nm (of the above) siRNA was performed by using Oligofectamine reagent (Invitrogen) according to the manufacturer's protocol. Eighteen hours after transfection the medium was changed to RPMI containing 2.5% FBS along with 100 nm nicotine for 48 h. After 48 h (on day 2), the transfection procedure was repeated. Eighteen hours post transfection the medium was changed to RPMI supplemented with 2.5% FBS along with 100 nm nicotine for 48 h. On day 4, lysates were made, and the relative expression of α7-nAChR and α3-nAChR was measured by ELISA. Each transfection was performed in duplicate, and the entire assay was performed two independent times.

##### Immunoprecipitation-Western Blotting

The co-immunoprecipitation (co-IP) experiments were performed by using the Mouse TrueBlot or the Rabbit TrueBlot kit (eBioscience, San Diego, CA) according to the manufacturer's protocols. Bound proteins were eluted in SDS sample buffer. The interaction of GATA4 (or GATA6) with Sp1 was probed by immunoblotting using standard protocols.

##### ChIP Assay

H520s were grown to 60% confluence in 100-mm tissue culture dishes. Subsequently, the medium was changed to RPMI with 2.5% FBS in the presence or absence of 100 nm nicotine for 48 h. Twenty-five million cells were used per ChIP reaction. The ChIP assay was performed as described previously (36). The differential binding of Sp1, GATA4 or GATA6 to the Sp1 binding sites on the human α7-nAChR promoter was examined by PCR using the primers listed in Mexal *et al.* (2010) (37). These primers were α7-nAChR-promoter-Sp1 forward (5′AGT ACC TCC CGC TCA CAC CTC G-3′) and α7-nAChR-promoter-Sp1 reverse (5′-ATG TTG AGT CCC GGA GCT GCA G-3′). The PCR was performed using PCR Mastermix (Promega) along with PCR enhancer (2.7 mm betaine, 6.7 mm DTT, 6.7% DMSO, 55 μg/ml BSA) (38). The PCR cycling conditions were 95 °C for 2 min, then 30 cycles of 95 °C for 30 s, 58 °C for 30 s, and 72 °C for 30 s followed by 72 °C for 2 min.

PCR for the region −267 to 37 bp of the human eukaryotic initiation factor 4E (eIF4E) promoter (which lacks Sp1 binding sites) was used as the negative control for the ChIP reaction (39). The primers used were eIF4E forward (5′-GTTGAGAACCGCGCACCCTACC-3′) and eIF4E reverse (5′-CACCGGTTCGACAGTCGCCATC-3′). The PCR cycling conditions were 95 °C for 2 min, then 30 cycles of 95 °C for 30 s, 65 °C for 30 s, and 72 °C for 3 min followed by 72 °C for 5 min.

##### ChIP-re-ChIP Assay

ChIP-re-ChIP assay was performed according to the protocol described by Pillai *et al.* (33, 40). The primary IP reaction was performed using monoclonal anti-human Sp1 antibody as described above. The immune complexes were eluted twice from the primary IP reaction by incubation with 10 mm DTT at 37 °C for 30 min. Subsequently the eluates were pooled, diluted 1:10 in with Re-ChIP buffer, and re-immunoprecipitated with 5 μg of GATA4 or GATA6 polyclonal antibody (33, 40). The protocol for preclearing and the IP reaction were identical to those described above for one-step ChIP assay (33, 40). The differential occupancy of the Sp1 binding sites on the human α7-nAChR promoter was analyzed by PCR as detailed above.

##### ChIP Assays with Human SCC-L Tumors

ChIP assays involving human SCC-L tumors from patients were performed using 2.5 mg of each tumor tissue per immunoprecipitation reaction. The ChIP assay was performed using the MAGnify Chromatin Immunoprecipitation System (Invitrogen) according to the manufacturer's instructions. The PCR for Sp1 sites on the α7-nAChR promoter and eIF4E was performed as described above.

##### Statistical Analysis

All data were represented as the mean ± S.E. (GraphPad Prism 5, La Jolla, CA). Results from the control and treated samples were compared using an analysis of variance followed by a Neumann-Keuls multiple comparison test. Those experiments involving only vehicle-treated and nicotine-treated samples were compared using a paired *t* test. All analyses were completed using a 95% confidence interval. Data were considered significant when *p* ≤ 0.05.

## RESULTS

### 

#### 

##### Nicotine Induces the Expression of α7-nAChRs on Human SCC-Ls Cell Lines and in Vivo

Previous studies have shown that multiple nAChR subunits are expressed on human SCC-L cell lines. Of the nAChR subtypes, the α7-nAChR mediates the proliferative effects of nicotine in human lung cancers. Results from our ELISAs show that the treatment of H520 cells with 100 nm nicotine induced a robust increase of α7-nAChR expression in a concentration-dependent manner (Fig. 1*A*, *black bars*) with the greatest expression of α7-nAChRs being observed from 100 nm to 10 μm at 96 h. This concentration range was chosen because nicotine concentrations in the plasma of average smokers range from 1 nm to 10 μm (6). In contrast, the level of the closely related α3-nAChR was minimally affected by nicotine treatment in H520 human SCC-L cells (Fig. 1*A*, *white bars*).

We also analyzed the time-kinetics of nicotine-induced α7-nAChR up-regulation in human SCC-Ls. We observed that the levels of α7-nAChR rise steeply at 96 h after nicotine treatment and remain relatively constant thereafter (data not shown). A nicotine concentration of 100 nm and the time point of 96 h were chosen for all subsequent experiments. Next we examined the effect of 100 nm nicotine on α7-nAChR expression in two additional human SCC-L cell lines, namely H226 and SK-MES (>96 h). ELISA experiments showed that nicotine induced a 2.5–3.0-fold increase of α7-nAChR levels in H226 and SK-MES human SCC-Ls over 96 h (Fig. 1*B*). However, nicotine did not have an impact on the expression of α3-nAChRs in H226 and SK-MES cells (data not shown).

Subsequently, we compared nicotine-induced α7-nAChR up-regulation to nicotine-induced proliferation of H520 human SCC-L cells. BrdU assays showed that the treatment of H520, H226, and SK-MES human SCC-L cells with 100 nm nicotine induced robust proliferation in these cell lines. The mitogenic activity of nicotine was reversed by the α7-nAChR antagonists α-bungarotoxin and methyllycaconitine, indicating that α7-nAChRs mediated the proliferative effects of nicotine on human SCC-Ls (Fig. 1*C*). We also observed that the concentration dependence and time-kinetics of α7-nAChRs up-regulation closely correlated with the proliferative effects of nicotine in human SCC-Ls (data not shown).

The next series of experiments aimed to examine whether nicotine could increase the expression of α7-nAChRs *in vivo*. The chicken CAM model was selected for these experiments. Several convergent studies have shown that human cancer cells implanted on chicken CAM grow as solid tumors and constitute an established model to measure tumor growth *in vivo* (31). H520 cells were implanted on chicken CAM and treated with 100 nm nicotine for 7 days. Nicotine potently accelerated the growth of H520 tumors in chicken CAM models (data not shown). Lysates were prepared from four untreated tumors (*white bars*, Fig. 1*D*) and four nicotine-treated tumors (*black bars*, Fig. 1*D*). ELISAs revealed that the H520 tumors treated with nicotine showed significantly higher levels of α7-nAChRs (*p* ≤ 0.05) than the untreated H520 tumors (Fig. 1*D*, *right panel*). In contrast, the expression levels of α3-nAChRs were unaffected by nicotine exposure (Fig. 1*D*, *left panel*). Therefore, nicotine-induced up-regulation of α7-nAChRs in human SCC-Ls was observed both in cell culture as well as in *in vivo* models.

Finally, the expression of α7-nAChRs was analyzed in human SCC-L tumors isolated from patients. Fig. 1*E* provides the smoking history of these patients. ELISAs show that the levels of α7-nAChR in tumors SCC-147, SCC-134, SCC-173, and SCC-104 (mean pack years smoked = 82) was significantly higher (*p* < 0.05) than that of SCC-103 and SCC-112 (mean pack years smoked = 45) (Fig. 1*F*, *black bars*). The levels of α3-nAChRs did not vary significantly in the SCC-L samples (Fig. 1*F*, *white bars*).

##### Nicotine Increases the Levels of α7-nAChRs mRNA in Human SCC-Ls

**FIGURE 2.**
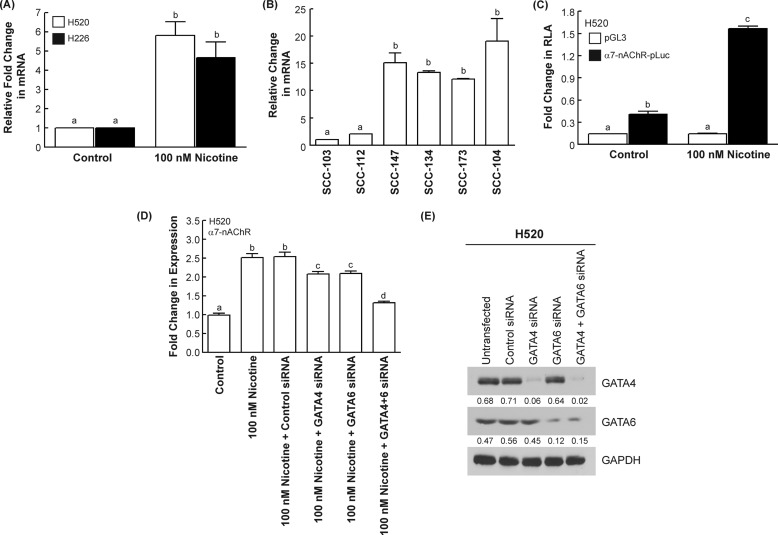
**Nicotine increased the transcriptional activity of α7-nAChR promoter in human SCC-L cells.** *A*, real-time PCR analysis revealed that the treatment of human SCC-L cell lines (H520 and H226) with 100 nm nicotine increased the levels of α7-nAChR mRNA in 72 h. *B*, the levels of α7-nAChR mRNA in human SCC-L tumors was correlated with the smoking history of the patients. *C*, luciferase reporter assays (*RLA*) demonstrated that nicotine induces luciferase activity in human SCC-L cells. H520 cells were transfected with the bovine α7-nAChR promoter construct, α7-promoter (bovine)-LUC. The addition of 100 nm nicotine potently increased nicotine-induced relative luciferase activity (firefly luciferase/renilla luciferase ratio) at 48 h. *D*, depletion of GATA4 and GATA6 by siRNA methodology reversed nicotine-induced up-regulation of α7-nAChR in H520 human SCC-L cells. The transfection of GATA4 siRNA (or GATA6 siRNA) individually had a minimal effect on the nicotine-induced increase of α7-nAChR (*fourth and fifth bar from the left*). However, the combination of GATA4 and GATA6 siRNA abrogated nicotine-induced increase in α7-nAChR expression. No effect was observed when the H520 human SCC-L cells were transfected with a control non-targeting siRNA. The figure represents the average of two independent experiments, and the data have been represented as the mean ± S.E. Results indicated by a different letter are significantly different (*p* < 0.05). *E*, Western blotting experiments revealed that the transfection of GATA4 and GATA6 siRNA suppressed the expression of these proteins in H520 cells. The Western blot was quantitated by densitometric analysis using NIH ImageJ 1.46p. The experiment was repeated twice, and the representative data are shown.

Real-time PCR analysis indicated that the treatment of H520 and H226 human SCC-L cells with 100 nm nicotine caused a 5–6-fold increase of α7-nAChR mRNA in both human SCC-L cell lines (Fig. 2*A*). Similarly, nicotine induced a 5-fold increase in α7-nAChR mRNA levels in H520 cells xenotransplanted on chicken CAM (data not shown). We performed a real-time PCR analysis of the human SCC-L tumors isolated from patients. We found that the α7-nAChR mRNA in tumors SCC-147, SCC-134, SCC-173, and SCC-104 (mean pack years smoked = 82; Fig. 2*B*) was substantially higher than tumors SCC-103 and SCC-112 (mean pack years smoked = 45; Fig. 2*B*). The levels of α7-nAChR mRNA correlated with the smoking history of these patients (Fig. 1*E*). These observations suggested that nicotine could be promoting α7-nAChRs levels by transcriptional mechanisms. This hypothesis was tested by transfection of bovine α7-nAChR-promoter-Luciferase into H520 cells as described under “Experimental Procedures.” Studies in bovine adrenal chromaffin cells have shown that the bovine α7-nAChR closely resembles the human α7-nAChR (94.6% identity at the protein level and 88% identity at the gene level). Also the bovine α7-nAChR promoter is similar to the human α7-nAChR promoter in terms of high G/C content, nature of transcription factor binding sites, and the location of these binding sites (11, 41). Several studies have used the bovine α7-nAChR promoter as a model for human α7-nAChR function. We observed that nicotine significantly increased α7-nAChR promoter activity, as evidenced by a 4-fold increase in relative luciferase activity relative to untreated control H520 cells (Fig. 2*C*). Nicotine did not induce any relative luciferase activity in mock-transfected H520 human SCC-Ls. We repeated the experiment in H226 human SCC-L cells and obtained similar results (data not shown).

Pioneering studies by Arredondo *et al.* (19) show a role for GATA2 in regulating α7-nAChR expression in human keratinocytes. With this background we decided to examine the role of GATA transcription factors (25) in nicotine-induced α7-nAChR up-regulation. Western blotting analysis showed that GATA1, -2, -3, and -5 were not expressed in H520, H226, and SK-MES human SCC-L cell lines. However, GATA4 and GATA 6 were robustly expressed in human SCC-Ls (data not shown).

The role of GATA4 and GATA6 in nicotine-induced α7-nAChR expression was examined by siRNA methodology. We found that the transfection of GATA4 siRNA or GATA6 siRNA (obtained from Santa Cruz Biotechnology) by themselves had only a marginal effect on nicotine-induced α7-nAChR expression. However, transfection of a combination of GATA4- and GATA6-siRNA abrogated nicotine-induced increase of α7-nAChR levels in H520 cells (Fig. 2*D*). This suggests that both GATA4 and GATA6 are important for nicotine-induced up-regulation of α7-nAChR in human SCC-Ls. Western blotting analysis showed that transfection of GATA4 siRNA and GATA6 siRNA efficiently decrease the expression of the GATA4 and GATA6, respectively, in H520 human SCC-L cells (Fig. 2*E*). These siRNA transfection experiments were repeated in H226 cells, and analogous results were obtained. The RNAi experiments were also repeated using a second set of independent GATA4 siRNA and GATA6 siRNA (purchased from Ambion Inc.), and similar results were obtained (data not shown). We observed that the transfection of GATA4 siRNA and GATA6 siRNA did not have any impact on the α7-nAChR (or α3-nAChR) expression in untreated control H520 and H226 cells (data not shown).

##### Nicotine Promotes the Binding of Sp1 and GATA4/GATA6 on the α7-nAChR Promoter in Human SCC-Ls

**FIGURE 3.**
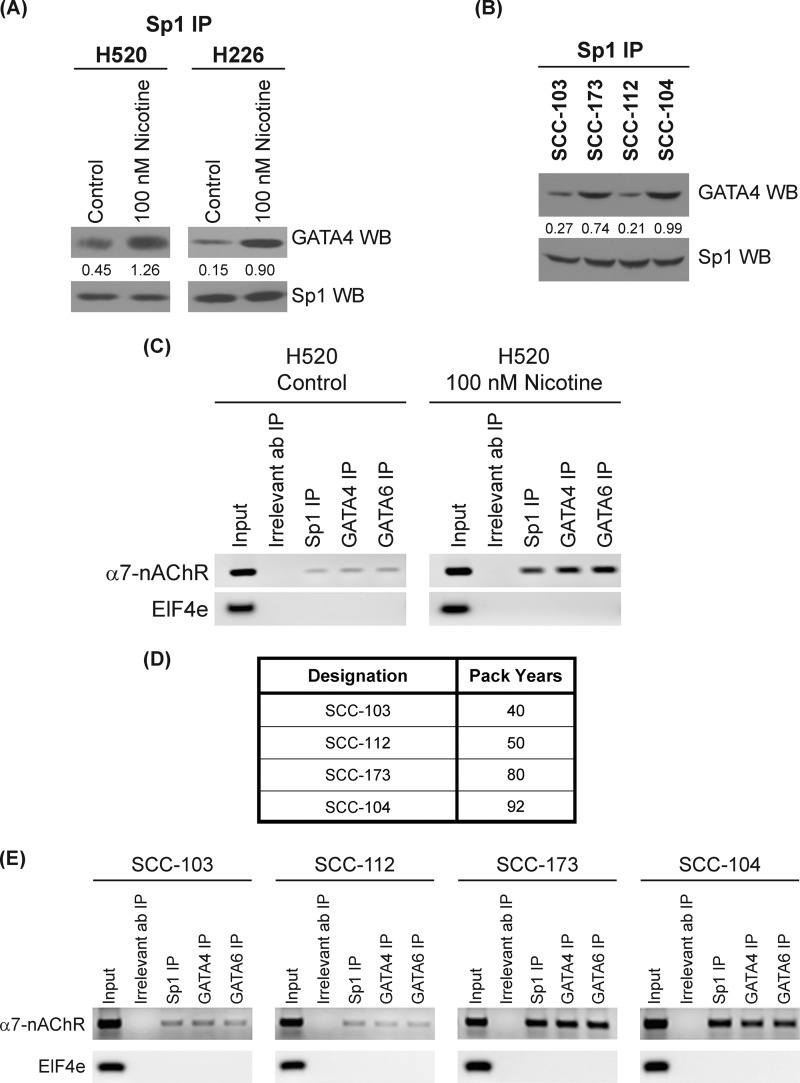
**Nicotine promoted the recruitment of Sp1, GATA4 and GATA6 to the α7-nAChR promoter in human SCC-Ls.** *A*, IP Western (*WB*) experiments showed that the treatment of H520 and H226 human SCC-L cells with 100 nm nicotine caused robust binding between Sp1 to GATA4. *B*, IP Western analysis of human SCC-L tumors showed that the level of Sp1-GATA4 complexes was closely correlated to the smoking history of the patients. The results were quantitated by densitometric analysis using NIH ImageJ 1.46p. The experiment was repeated twice, and the representative data are shown. *C*, ChIP assays demonstrated that nicotine increased the amount of Sp1, GATA4, and GATA6 on the human α7-nAChR promoter in nicotine-treated H520 human SCC-L cells. Untreated H520 cells contained low levels of the amounts of Sp1, GATA4, and GATA6 associated with the α7-nAChR promoter (*top lane*, *left panel*). The treatment of H520 cells with 100 nm nicotine robustly increased the levels of Sp1, GATA4, and GATA6 bound to the α7-nAChR promoter (*top lane*, *right panel*). PCR for a region of the EIF4e promoter (lacking Sp1 sites) was taken as the control for the experiment (*bottom panel*). *D*, a table showing the human SCC-L tumors used for ChIP assay. *E*, ChIP assays on the human SCC-L tumors show that the amounts of Sp1, GATA4, GATA6 on the human α7-nAChR promoter in SCC-103, and SCC-112 (mean pack years smoked = 45) was substantially lower that the levels of Sp1, GATA4, and GATA6 bound to the human α7-nAChR promoter in SCC-173 and SCC-104 (mean pack years smoked = 86). The *Input* DNA lane represents one-fifth of the precleared chromatin used in each ChIP reaction (*left panel*). The figure is representative of two independent experiments.

Previous literature has shown that Sp1 can directly bind to both GATA4 and GATA6 in response to extracellular stimuli and regulate gene expression (42, 43). We wanted to assess the effect of nicotine on the interaction between Sp1, GATA4, and GATA6. Because the molecular weight of GATA4 and GATA6 is close to the molecular weight of the IgG heavy chain (both 50 kDa), we decided to use the TrueBlot IP Western system (eBiosciences Inc., San Diego, CA) for the IP-Western blot experiments. The TrueBlot kit masks the IgG band; therefore, proteins near 50 kDa are easily visualized. An IP-Western blot revealed that serum-starved H520 cells contained very little binding between Sp1 and GATA4. The treatment of H520 cells with 100 nm nicotine led to a robust binding between Sp1 and GATA4 at 48 h (Fig. 3*A*, *left panel*). We repeated the IP-Western experiments with a second cell line H226 and obtained similar results (Fig. 3*A*, *right panel*). Nicotine also induced the binding of Sp1 to GATA6 in both H520 and H226 cells in 48 h (data not shown). We repeated the IP-Western blot the other way around. We immunoprecipitated H520 cell lysates with GATA4 (or GATA6 antibody) and probed the presence of Sp1 in the immunoprecipitates by Western blotting. We obtained comparable results as depicted in Fig. 3*A* (data not shown).

The interaction of Sp1 with GATA4 and GATA6 was also analyzed in the human SCC-L tumors isolated from patients. We observed that the tumors SCC-103 and SCC-112 (mean pack years = 45) displayed lower Sp1-GATA4 binding than the tumors SCC-104 and SCC-173 (mean pack years = 86), which had a higher exposure to nicotine (Fig. 3*B*). We also observed a similar pattern in the binding of Sp1 and GATA6 in the human SCC-L tumors (data not shown). Our data suggests that the exposure of human SCC-L cells to nicotine potently induced the binding of Sp1 to GATA4 or GATA6.

ChIP assays were performed to examine the relative distribution of Sp1, GATA4, and GATA6 on the human α7-nAChR promoter in human SCC-L cells. Data by Gault *et al.* (22) have demonstrated the presence of five Sp1 sites on the human α7-nAChR promoter in the region ranging from −1 to −202. Gault *et al.* (22) and Leonard *et al.* (23) have labeled the translation start site as −1, and the sequences upstream are labeled in increasing order accordingly (22, 23). ChIP assays revealed that untreated H520 cells contained low levels of Sp1, GATA4, and GATA6 bound to the α7-nAChR promoter (Fig. 3*C*, *left panel*, *top lane*). The presence of 100 nm nicotine resulted in robust amounts of Sp1, GATA4, and GATA6 bound to the α7-nAChR promoter (Fig. 3*C*, *right panel*, *top lane*). No signal was obtained with the irrelevant antibody (donkey anti-rabbit IgG; Fig. 3*C*). PCR for a region of the eIF4E that contains no Sp1 sites was used as a negative control for the experiment (Fig. 3*C*, *bottom lane*).

We performed the ChIP assay in four of the human SCC-L tumor samples (Fig. 3*D*). The tumors SCC-104 and SCC-173, which were exposed to nicotine for a longer time, showed greater amounts of Sp1, GATA4, and GATA6 bound to the α7-nAChR promoter (Fig. 3*E*; *top row*, *rightmost two panels*) relative to tumors SCC-103 and SCC-112 (which were exposed to lower amounts of nicotine) (Fig. 3*E*; *top row*; *leftmost two panels*). PCR for the eIF4E promoter region (lacking Sp1 binding sites) was used as a negative control for the experiment (Fig. 3*E*, *bottom lane*).

**FIGURE 4.**
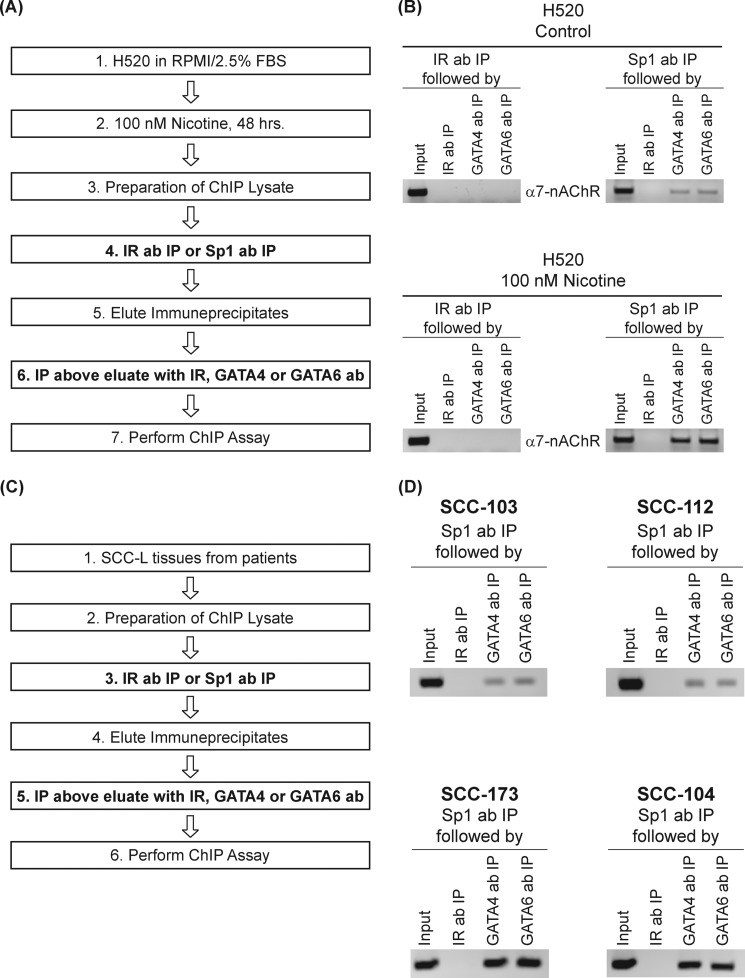
**ChIP-re-ChIP assays reveal increased Sp1-GATA4 and Sp1-GATA6 complexes on the human α7-nAChR promoter in nicotine-treated H520 cells and human SCC-Ls.** *A*, a flowchart describing the sequential steps involved in ChIP-re-ChIP experiment in H520 cells. *B*, untreated H520 control cells had very low amounts of Sp1-GATA4 and Sp1-GATA6 complexes on the α7-nAChR promoter (*top right panel*). The onset of nicotine treatment led to robust amounts of Sp1-GATA4 and Sp1-GATA6 complexes on the α7-nAChR promoter (*bottom right panel*). The antibodies used the second round of chromatin IP, namely GATA4 polyclonal antibody and GATA6 polyclonal antibody, are indicated vertically next to the individual lanes. *C*, a flowchart describing the sequential steps involved in ChIP-re-ChIP experiment in human SCC-L tumors (isolated from patients). *D*, SCC-103 and SCC- 112 human SCC-L tumors had lower levels of Sp1-GATA4 and Sp1-GATA6 complexes bound to the α7-nAChR promoter (*top panels*) compared with SCC-173 and SCC-104 human SCC-L tumors (*bottom panels*). The input DNA lane represents one-fifth of the precleared chromatin used in each ChIP reaction. The figure is representative of two independent experiments.

We further examined whether nicotine could induce the recruitment of Sp1-GATA4 or SP1-GATA6 complexes α7-nAChR promoter. Therefore, we performed ChIP-re-ChIP on these promoters using the Sp1 antibody. Quiescent and nicotine-treated H520 ChIP lysates were immunoprecipitated with Sp1 antibody to bring down all the promoter elements bound to Sp1. The schema of the assay is shown in Fig. 4*A*. Rabbit-anti-mouse IgG was used as the irrelevant antibody (IR ab) for the first round of chromatin immunoprecipitations (Fig. 4*A*, *step 4*). These Sp1-bound chromatin complexes were eluted and re-immunoprecipitated with antibodies to GATA4 or GATA6 to isolate the complexes containing both Sp1 and GATA4 (or Sp1 and GATA6). Donkey-anti-rabbit IgG was used as the IR ab for the second round of chromatin immunoprecipitations (Fig. 4*A*, *step 6*). PCR was performed using the second immunoprecipitated DNA to amplify fragments of the α7-nAChR promoter.

ChIP-re-ChIP revealed that untreated control H520 cells had very low binding between Sp1-GATA4 and Sp1-GATA6 proteins on the α7-nAChR promoter (Fig. 4*B*, *top right panel*). The treatment of H520 cells with 100 nm nicotine for 48 h resulted in increased binding of Sp1 to GATA4 and Sp1 to GATA6 (Fig. 4*B*, *bottom right panel*). No signal was obtained with the irrelevant antibody (Fig. 4*B*, *top* and *bottom left panels*) in the ChIP-re-ChIP assay.

Subsequently, we performed a ChIP-re-ChIP assay in human SCC-L tumors (Fig. 3*D*) isolated from patients. The schema of the assay is outlined in Fig. 4*C*. Rabbit-anti-mouse IgG was used as the IR ab for the first round of chromatin immunoprecipitations (Fig. 4*C*, *step 3*). Donkey-anti-rabbit IgG was used as the IR ab for the second round of chromatin immunoprecipitations (Fig. 4*C*, *step 5*). We observed that tumor SCC-173 and SCC-104 (mean pack years smoked = 86) contained high amounts of Sp1-GATA4 and Sp1-GATA6 complexes bound to the α7-nAChR promoter (Fig. 4*D*, *bottom two panels*), whereas the tumors SCC-103 and SCC-112 (mean pack years smoked = 45) contained lower amounts of Sp1-GATA4 and Sp1-GATA6 complexes associated with the α7-nAChR promoter (Fig. 4*D*, *top two panels*). No signal was obtained using the irrelevant antibody in the ChIP-re-ChIP assay (data not shown).

We performed a chromatin IP in the reverse fashion using GATA4 polyclonal antibody followed by ChIP-re-ChIP to confirm these results (data not shown) in human SCC-L tumors. Taken together our data suggest that exposure to nicotine promoted the recruitment of Sp1-GATA4 and Sp1-GATA6 complexes to the α7-nAChR promoter. This event induced the transcription of α7-nAChR and increased its levels in human SCC-L cells exposed to nicotine.

## DISCUSSION

The homomeric α7-nAChR subtype is responsible for the proliferative, pro-angiogenic and prometastatic activities of nicotine in lung cancers (6, 7, 10). Several research studies have examined the molecular mechanisms by which α7-nAChR promotes the progression of human lung cancers. The α7-nAChR has been shown to cause direct activation of inactivation of signaling kinases and phosphatases. In addition, nicotine induces epithelial-to-mesenchymal transition and metastasis of human lung cancers in a α7-nAChR-dependent manner (6, 7). Therefore, it may be envisaged that lung tumors with high levels of α7-nAChR may be highly vascularized and prone to metastasis.

The present study shows that exposure to nicotine increases the levels of α7-nAChRs in human SCC-Ls. The up-regulation of α7-nAChR was found to occur at concentrations of nicotine present in the plasma of an average smoker (1 nm to 10 μm) (44). Exposure to cigarette smoke and nicotine has been shown to increase the levels of α7-nAChRs in several types of non-neuronal cells like human monocytes, keratinocytes, normal lung epithelial cells, and pancreatic ductal adenocarcinoma tumors (10, 19, 45, 46). Arredondo *et al.* (19) observed that the nicotine up-regulated α7-nAChR expression in human keratinocytes in a similar manner as environmental tobacco smoke. These findings suggest that the effect of nicotine on α7-nAChR expression closely resembles that of cigarette smoke. We also found that the levels of α3-nAChR did not change substantially upon nicotine treatment. This is in agreement with previous data that long term nicotine exposure has an diverse impact on the activity of different nAChR subtypes (14). In particular, the levels of α3-nAChR have been found to remain relatively unchanged upon exposure to nicotine in non-neuronal cells (19). However, it must be remembered that the nicotine may regulate the expression of nAChR subunits in a tissue-dependent manner.

We also observed that nicotine increases the levels of α7-nAChR by transcriptional mechanisms. This is in contrast with data from neuronal cells where nicotine-induced up-regulation of nAChR subunits has been found to occur via posttranslational mechanisms (14). These mechanisms include alteration in nAChR assembly, maturation, turnover, and inhibition of receptor degradation. However, a majority of these papers studied the effect of nicotine on α2, α3, α5, and α4/β2-nAChR subunit mRNA in mouse and rat brains (14). Several lines of evidence suggest that the effect of nicotine on α7-nAChR expression and function differs from those of heteromeric nAChRs (16, 27). It also may be probable that the mechanisms underlying nicotine-induced up-regulation of α7-nAChR may be different in non-neuronal cells than in the brain.

We also analyzed the mechanisms by which nicotine increases transcription of α7-nAChRs. Based on the results of Arredondo *et al.* (19) we analyzed the role of the GATA family of proteins in nicotine-induced up-regulation of α7-nAChR. The α7-nAChR promoter has several binding sites for Sp1 (21–23). Previous studies have demonstrated that the Sp1 protein can directly associate with the GATA family of transcription factors to regulate gene expression (25). Of the GATA family of transcription factors GATA1, GATA2 and GATA3 are predominantly hematopoietic and were not found in human SCC-Ls (25). Similarly, GATA5 was not detected in human SCC-L cell lines. Clinical studies have indicated that GATA4 and GATA6 are robustly expressed in SCC-L cell lines and tumors (47, 48). The transcription factor Sp1 can interact directly with GATA4 and GATA6 in a signal-dependent manner in several experimental systems to regulate gene expression (42, 43). Our results provide yet another example where nicotine induces the binding of GATA4 or GATA6 to Sp1 on the α7-nAChR promoter. This process increased α7-nAChR transcription and expression in SCC-Ls. A comparison of Sp1-GATA4 and Sp1-GATA6 complex levels between SCC-L patients who are smokers *versus* non-smokers would have been ideal. However, SCC-Ls are rare in non-smokers.^5^ Therefore, we were unable to perform this comparison in our study.

Epidemiologic studies have suggested that cumulative cigarette smoking (measured in pack-years) or current cigarette smoking status is related directly to the clinical prognosis of lung cancer patients (29, 49, 50). Our findings provide a potential molecular mechanism for these clinical observations in human SCC-Ls. Our future studies aim to examine whether nicotine-induced up-regulation of α7-nAChR is a single-step process or involves multiple steps. Data from Arredondo *et al.* (19) show that nicotine induced the sequential up-regulation of α5-nAChR and α7-nAChR in human keratinocytes. It may be possible that such a multistep process also exists in human SCC-Ls. Our manuscript has only studied the effect of nicotine on α7-nAChR levels. It is possible that nicotine regulates several nAChR subunits to promote the overall survival of lung tumors.

One of our future research objectives is to identify the signaling kinases that mediate nicotine-induced up-regulation of α7-nAChR in human SCC-Ls. The α7-nAChR promoter has multiple Sp1 sites. We aim to analyze whether all these Sp1 sites are involved in nicotine-induced α7-nAChR up-regulation or perhaps some of the Sp1 sites are more important than the others. We believe such studies will uncover new knowledge about the role of nicotine-nAChR-signaling pathway in human SCC-Ls.

## Footnotes

5 J. F. Pulliam, personal communication.

## Footnotes

4 The abbreviations used are:

SCC-L: squamous cell carcinoma of the lung
nAChR: nicotinic acetylcholine receptor
CAM: chorioallantoic membrane
IP: immunoprecipitation
eIF4E: eukaryotic initiation factor 4E
IR ab: irrelevant antibody.
